# Simulated medical information system: education for aspiring healthcare information technologists

**DOI:** 10.20407/fmj.2021-022

**Published:** 2022-05-25

**Authors:** Koichi Muto, Soichiro Koyama, Shigeo Tanabe, Hiroaki Sakurai, Yoshikiyo Kanada

**Affiliations:** 1 Faculty of Medical Management and Information Science, Fujita Health University, School of Medical Sciences, Toyoake, Aichi, Japan; 2 Faculty of Rehabilitation, Fujita Health University, School of Health Sciences, Toyoake, Aichi, Japan

**Keywords:** Simulation materials, Healthcare information technologies, Education

## Abstract

**Objectives:**

To determine if a simulated medical information system can improve the level of understanding of healthcare information technology students.

**Methods:**

The study involved 40 healthcare information technology students. All the students took the healthcare information technology course using the simulated medical information system. The primary outcome was a measure of their level of understanding assessed with a questionnaire using a five-point Likert-type scale. The questions were all included in the required knowledge for the Specific Behavioral Objectives for Healthcare Information Technologists (2016) and Senior Healthcare Information Technologists (ver. 1.1, 2017). To measure the level of understanding, median with 10th–90th percentile CI values for both sets of questionnaires were calculated for all the students. The Wilcoxon signed-rank test was used to compare level of understanding before and after the training.

**Results:**

Some students were excluded because they failed to complete the questionnaires. For both Healthcare Information Technologists (n=37) and Senior Health Information Technologists (n=34), the level of understanding was significantly different before (median [10th–90th percentile]: 1175 [935–1271], 416 [302–513]) and after (1200 [1016–1472], 469.5 [351–527]) the training (p<0.05).

**Conclusions:**

A simulated medical information system may be an effective tool for students to learn about healthcare information technology.

## Introduction

Information and communication technology (ICT) now plays a significant role in healthcare services, ensuring efficiency, quality improvement, safety, medical cost containment, and transparency.^1^ Medical information systems (MISs) and electronic medical record (EMR) systems are now widely used, not only in Japan but also worldwide.^2^ However, flexible system improvement has been difficult because there are few experts in the field of healthcare information technology. Training Healthcare Information Technologists (HITs) has therefore been recognized as pivotal in healthcare. The digitization of health information, resulting in more diverse and complex healthcare information,^3^ has prioritized the need for HITs with the requisite knowledge and skills. Institutions of higher learning need to provide efficient and practical education to meet this need.

The HIT Certification Program, authorized by the Japan Association for Medical Informatics (JAMI), was implemented in 2003 to educate students in healthcare information technology.^4,5^ Many professionals in healthcare and industry, including software engineers, managers, and vendors involved in healthcare information technology, are encouraged to obtain this certification. It provides the required knowledge and skills related to information technology, healthcare, and MISs. HIT certification shows that an individual can effectively “communicate, collaborate, and coordinate”.^4,5^ Students aspiring to be an HIT need to clearly understand how MISs work and be able to interconnect (and integrate) those systems and subsystems in very practical ways. EMR systems facilitate healthcare integration and interoperability, but commercial EMR systems are difficult to use for student education because of system policies/procedures and problems with ease of use.

This study therefore aimed to clarify if students’ level of understanding is improved by using a simulated medical information system (SMIS) as part of a simpler, user-friendly course in the field of healthcare information technology.

## Methods

### Participants

The study involved 40 students from the faculty of Medical Management and Information Science, School of Health Sciences, Fujita Health University. All were third-year university students who had taken all the credits (23) for both the information science courses (e.g., information processing, database, network technology, information security, and programming) and the medical information courses (e.g., medical informatics and hospital information system studies). All were informed of the study procedures for participation and provided written and verbal consent. The study protocol was approved by the Human Ethics Committee of the University (No. HM19-277).

### SMIS

**Figure 1:**
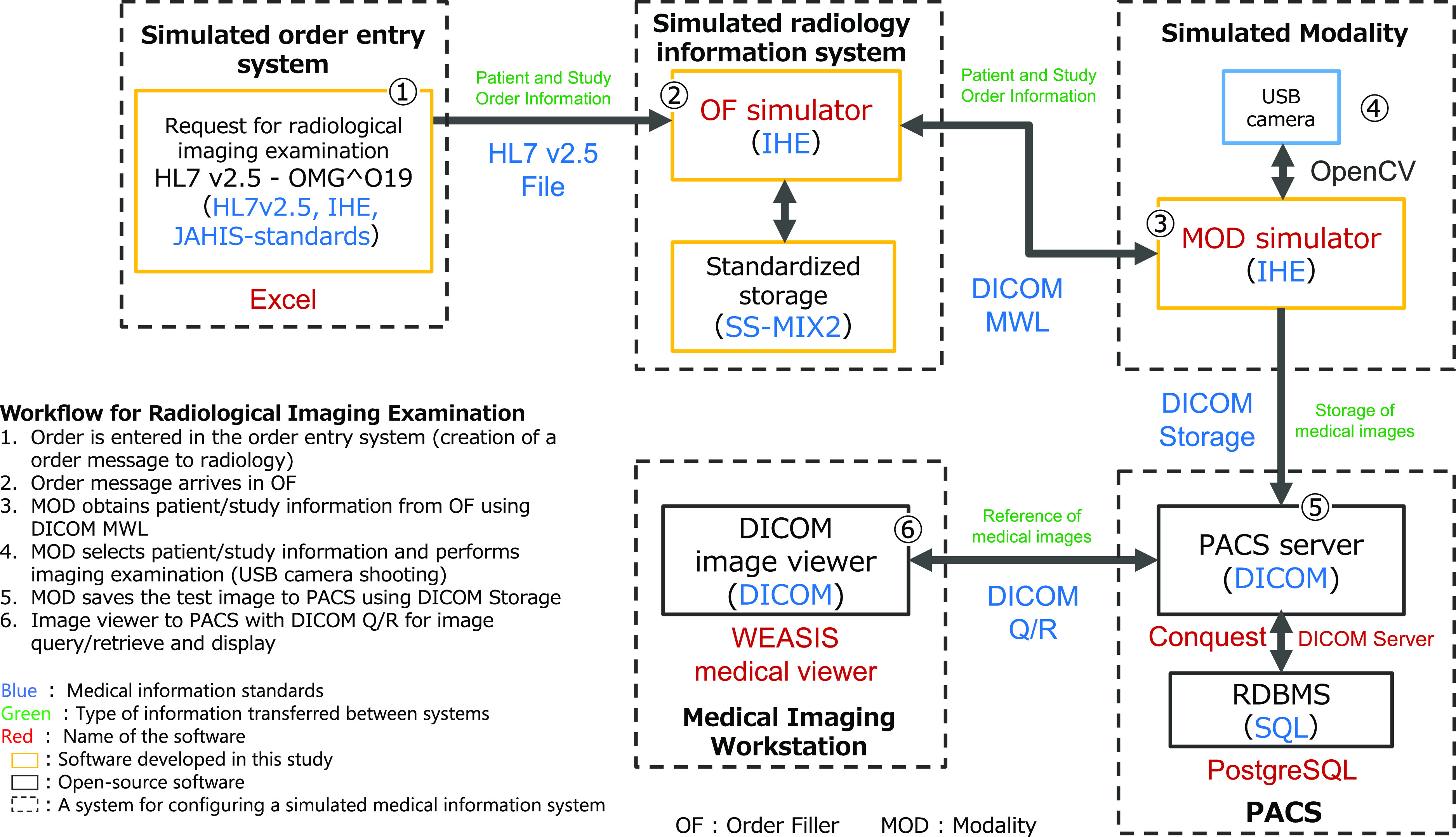
The system diagram of the simulated medical information system (SMIS) used in this study.

The SMIS used in this study can simulate actual processes used in the medical professions division system, including the radiology department. These processes include ordering and performing a radiological examination and saving the images. Using the SMIS, students can learn to implement, configure, and operate systems and configure data linkage. This system takes full advantage of the use of open-source software, which is integral to the function of the MISs. The system is cross-platform (Java and JavaScript) without operating system dependence. And the intersystem coordination was incorporated in line with medical information standards. The system diagram of the SMIS used in this study is shown in Figure 1. The SMIS is designed to act as teaching material that enables students to understand the internal structure (architecture) of an information system, and to learn how to set up and operate an information system correctly through personal experience. It was built entirely using open-source software. An information system consists of multiple subsystems, and data are sent and received among subsystems to exchange essential information to support the operation of the system. Information systems in medicine have a core Order Entry System, which transmits order data to the departmental system. The information in the order data is further transmitted from the departmental system to the medical engineering equipment and other departmental systems. The SMIS simulates the information system for the radiology department, which is one of the most standardized systems in medicine. The workflow of a radiology examination is shown in the following order: imaging examination order information (including patient information such as patient ID and name, and study information such as imaging examination method) entered in the Order Entry System is transmitted to the Radiology Information System (RIS) in Health Level Seven (HL7) V2.5^6^ format. The transmitted imaging examination order information is then transmitted from the RIS to the modality system (modality) in the form of Digital Imaging and Communications in Medicine (DICOM)^7^ Modality Worklist (MWL), and information such as the patient ID is given as accompanying information for the captured image. The image data with accompanying information are transmitted in DICOM image format to the Picture Archiving and Communication System (PACS) for archiving. The stored image data are searched by the DICOM Image Viewer using the DICOM Query/Retrieve (Q/R) format for images stored in the PACS using keywords in the accompanying information, and the corresponding images are transferred from the PACS to the image viewer. The transferred images are displayed in the image viewer, and supplementary information such as patient ID can be confirmed. For the SMIS, we did not create an Order Entry System, but instead used an Excel sheet that generates HL7 V2.5 OMG O19-type clinical order messages in line with the Japanese Association of Healthcare Information Systems Industry (JAHIS) standard,^8^ which is the imaging examination order information. Subsystems corresponding to PACS and DICOM image viewer were adapted from those available on the Internet as open-source software. The RIS and Modality simulators are newly implemented and named the OF simulator and MOD simulator. The OF simulator simulates the functions of Order Filler, as defined in Integrating the Healthcare Enterprise (IHE),^9^ and the MOD simulator simulates some of the functions of Modality in IHE.^9^ For each subsystem, the user interface and application logic were implemented using a server-side JavaScript environment (node.js^10^) so that the internal structure of the subsystem can be easily checked. To handle HL7 V2.5 and DICOM in each subsystem, we used the open-source software libraries (HAPI^11^, dcm4che^12^) created by Java. The OF simulator receives HL7 V2.5 text files as radiology study orders and stores them in SS-MIX2 Standardized Storage.^13^ Usually, a Relational DataBase Management System (RDBMS) is used to manage the information stored in the information system. However, using an RDBMS requires considerable man-hours to set up and manage the system, which makes the exercise more complicated. The use of SS-MIX2 provided an opportunity for practical experience of this approach, which is one of the national standards set by the Ministry of Health, Labour and Welfare of Japan.^14^ The MOD simulator controls the USB camera using the open-source software visualization library (OpenCV^15^), creates the still image data from the USB camera video, and obtains patient and study information from the OF simulator using DICOM MWL. The MOD simulator then converts the still images and the patient and study information into DICOM-format image data. We implemented a function in the MOD simulator to send the generated DICOM image data to PACS using the DICOM storage protocol.

### Experimental procedure

All the students learned about health information technology using the SMIS. A certified Health Information Technologist (HIT) provided lectures and practical training to give the students basic knowledge about the SMIS. There were two 90-minute lectures providing an overview and a description of the SMIS, and the simulation of the actual work of an HIT. There were also two 90-minute practical training sessions that discussed the installation, configuration, and execution of the SMIS. After each student’s ability to use the SMIS competently had been confirmed, they could then use it to execute the practical duties of an HIT. The SMIS class was conducted as follows. As an orientation, students were given an overview of SMIS and the technologies used in its implementation. Hands-on exercises were conducted using the SMIS installation and configuration manual, including correct installation and configuration of each subsystem of SMIS. The participants were also asked to check that information was transmitted among subsystems and that order information contained in HL7 V2.5 text files created with Excel sheets was stored as accompanying information for images displayed in the Image viewer. The questionnaires to assess level of understanding of the course content were administered before and after the lectures and practical training.

### Outcome

The primary outcome measure was students’ level of understanding, assessed via two sets of questionnaires using a five-point Likert-type scale. Each set of questions covered all items in the Specific Behavioral Objectives for either HITs or Senior HITs (ver.1.1), which were developed as part of the JAMI HIT Certification.^16,17^ The total number of items was 396 for HITs and 171 for Senior HITs. The HIT knowledge and skills included a comprehensive set of content required for medical information technicians. Certification for Senior HITs also emphasizes management skills. Only the Japanese version of this questionnaire has been published; the official English version has not been published. All students were asked to document their level of understanding on a five-point scale for both sets of questionnaires before and after the lectures and practical training.

### Statistical analysis

Summed self-scores were compared before and after the training. To measure the level of understanding, median with 10th–90th percentile CI values for both sets of questionnaires were calculated for all the students. The Wilcoxon signed-rank test was used to compare the values before and after the training. All statistical analyses used SPSS (version 25; IBM, Armonk, NY, United States), and statistical significance was set at a p-value of 0.05.

## Results

**Figure 2:**
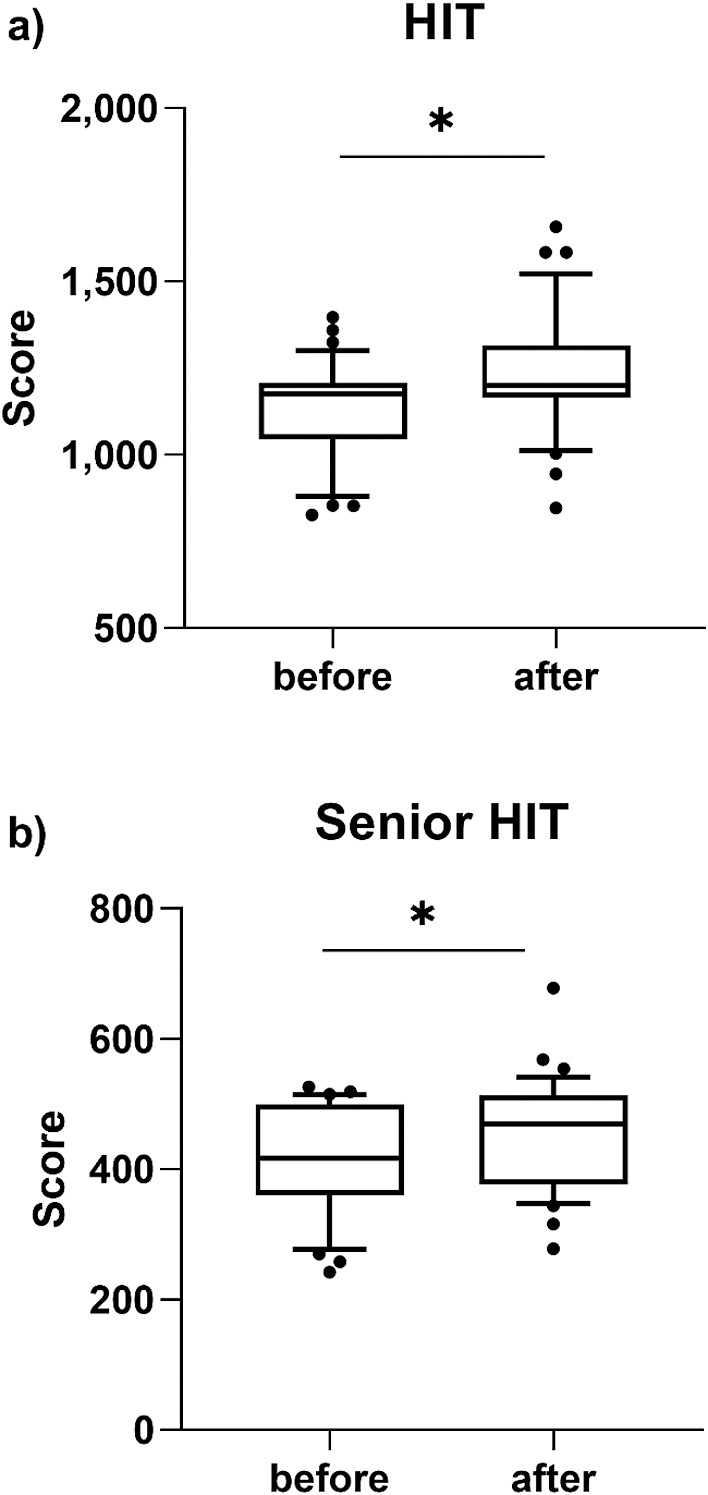
Level of understanding before and after training. a) Questions based on Healthcare Information Technologist: Specific Behavioral Objectives (2016). b) Questions based on Senior Health Information Technologist: Specific Behavioral Objectives ver. 1.1 (2017). Data shown as median on 10th to 90th percentile, p<0.05.

Three students were excluded because they failed to complete the questionnaires on the HIT requirements, and a further six because they failed to complete the questionnaires on the Senior HIT requirements. In the analysis of the knowledge required for HITs, there was a significant difference in the level of understanding before (median [10th–90th percentile]: 1175 [935–1271]) and after (1200 [1016–1472]) the training (*p*<0.05). There was also a significant change in the level of understanding of the knowledge required for a Senior HIT after training (416 [302–513] before vs. 469.5 [351–527] after, *p*<0.05) (Figure 2).

## Discussion

In this study, we aimed to clarify the effect of an SMIS-based HIT curriculum on students’ level of understanding after course completion. The results indicate that the SMIS may significantly improve the level of understanding of the knowledge required for both HIT and Senior HIT certification, and may therefore be an effective tool in HIT-related education.

The specific behavioral objectives for HITs cover detailed knowledge and skills in three areas: information technology, healthcare, and MISs. In this study, the SMIS-based lectures and practical training were purposefully designed to provide diverse opportunities to learn about these subject areas and develop the required behavioral objectives.

The behavioral objectives for Senior HITs include the knowledge and skills required for HITs in upper-level management positions to facilitate problem-solving, management, advanced data analysis, technical thinking and communication, ICT use, information security, human resource development, and a diverse knowledge of health information systems. Many of the specific behavioral objectives for Senior HITs focus on managerial competencies, but some cover upper-level knowledge/skills in the areas of information technology and MISs. A simulation may therefore improve the level of understanding in these areas.

In previous studies, practical training has proved to be more effective than lectures for knowledge consolidation.^18^ Bay et al. reported that discovery learning was better than direct instruction for optimum knowledge retention.^19^ The SMIS simulates healthcare information processing tasks required in a healthcare setting. Using these simulations therefore provides for immediate application of knowledge learned in lectures, improving both level of understanding and retention. The SMIS also allows students to understand and practice system improvement, internal changes, and the adjustment of data linkage, all of which are difficult to perform with commercial EMRs. This is supported by the literature. For example, Cyr et al. reported that trial-and-error learning led to superior source memory than errorless learning.^20^ Experiencing errors and developing a plan to address them via the SMIS may also facilitate knowledge retention.

We believe that teaching using SMIS can promote understanding of several elements, including architecture of information systems, installation and configuration of information systems for proper operation, methods of transferring information between information systems and standard technologies used for the transfer (standardization). These are related to the knowledge items in the field of medical information systems and information processing technology associated with the expertise of HITs.

Conventional curricula only allow students to understand the specifications and grammar of programming languages, and to create programs to solve simple problems. We believe that the SMIS-based classes can promote understanding of the internal structure (architecture) of information systems through experiential learning of how to install and configure information systems and make them work properly. We also believe that it is better to allow students to use their hands and learn through experience, rather than teaching the architecture of the information system and the method of setting up the operation using lectures, which only explain the concept. This is particularly true for students who are close to first-time learners. It would also be possible to run a class where students program and build the information system itself from scratch. In a class using SMIS, students are required to prepare and install the libraries and middleware necessary to run each subsystem of the SMIS, as well as to install and configure the subsystems themselves. The students will also experience the proper installation and configuration of the subsystems according to the manual. HITs are required to have the skills to operate and manage information systems correctly, rather than the programming skills to build information systems from scratch. We believe that classes using SMIS will provide students with the starting point of the knowledge that they need to acquire “skills to operate and manage information systems properly”, which has been difficult to teach in the past.

There is a limitation in this study, as it was unblinded and uncontrolled. It is ethically difficult to use a non-education group as a control group for educational research, but blinding the raters and increasing the sample size would have provided better evidence to determine the effectiveness of an SMIS-based HIT education to mitigate the limitations of this study. We used the convenience sampling method to find participants from among third-year university students who had taken all the credits for both the information science courses and the medical information courses. We therefore did not carry out a sample size estimation before the experiment. For a future randomized control study, sample size estimation could be used to estimate the effect size of SMIS based on our results. We also included only one year-group of students as participants. To confirm the generalizability of the results, it would be helpful to carry out a multidimensional evaluation of the effect on academic performance in students from this and other years. This study did not conduct an objective evaluation of the knowledge items that we assume can be taught by SMIS. Future research is needed to develop an evaluation method to measure the level of understanding of knowledge items. We believe that the effectiveness of SMIS education will become clearer if a two-way evaluation is conducted (an evaluation from the teacher’s side using an examination related to SMIS education, as well as the evaluation from the students).

## References

[1] Chaudhry B, Wang J, Wu S, Maglione M, Mojica W, Roth E, Morton SC, Shekelle PG. Systematic review: impact of health information technology on quality, efficiency, and costs of medical care. Ann Intern Med 2006; 144: 742–752. 16702590 10.7326/0003-4819-144-10-200605160-00125

[2] Kanakubo T, Kharrazi H. Comparing the trends of electronic health record adoption among hospitals of the United States and Japan. J Med Syst 2019; 43: 224. 31187293 10.1007/s10916-019-1361-y

[3] Brenner SK, Kaushal R, Grinspan Z, Joyce C, Kim I, Allard RJ, Delgado D, Abramson EL. Effects of health information technology on patient outcomes: a systematic review. J Am Med Inform Assoc 2016; 23: 1016–1036. 26568607 10.1093/jamia/ocv138 PMC6375119

[4] Information on IMIA Regional Groups. Yearb Med Inform 2015; 256–271.

[5] Kawamura T, Hashimoto N, Ishikawa K. A New Specialist in the Area of Medical Informatics: Healthcare Information Technologist. Japan Journal of Medical Informatics 2003; 23: 431–440 (in Japanese).

[6] Health Level Seven International. HL7 Messaging Standard Version 2.5; 2003. <https://www.hl7.org/implement/standards/product_brief.cfm?product_id=143> (Accessed October 12, 2021).

[7] The Medical Imaging & Technology Alliance (MITA), a division of the National Electrical Manufacturers Association (NEMA). Current Edition—the DICOM Standard; 2021. <https://www.dicomstandard.org/current> (Accessed October 12, 2021).

[8] Japanese Association of Healthcare Information Systems Industry (JAHIS). JAHIS housysen data koukan kiyaku Ver.3.1.C (Radiology Data Exchange Standard Ver.3.1.C issued by JAHIS); 2017 (in Japanese). <https://www.jahis.jp/files/user/04_JAHIS%20standard/17-002_JAHIS%E6%94%BE%E5%B0%84%E7%B7%9A%E3%83%87%E3%83%BC%E3%82%BF%E4%BA%A4%E6%8F%9B%E8%A6%8F%E7%B4%84%20Ver.3.1C.pdf> (Accessed October 12, 2021).

[9] Integrating the Healthcare Enterprise International, Inc. IHE Radiology Technical Framework. Volume 1 Revision 19.0; 2020. <https://www.ihe.net/uploadedFiles/Documents/Radiology/ IHE_RAD_TF_Vol1.pdf> (Accessed October 12, 2021).

[10] OpenJS Foundation. Node.js. <https://nodejs.org/en/> (Accessed October 12, 2021).

[11] University Health Network. HAPI project; 2017. <https://hapifhir.github.io/hapi-hl7v2/> (Accessed October 12, 2021).

[12] dcm4che project. dcm4che.org. <https://www.dcm4che.org/> (Accessed October 12, 2021).

[13] Japan Association for Medical Informatics. “SS-MIX2 Standardized Storage” explanation of the structure and guidelines for inplemenation Ver.1.2; 2014. <https://www.jami.jp/jamistd/docs/SS-MIX2/descript-implemglonSS-MIX2_V1.2.pdf> (Accessed October 12, 2021).

[14] Ministry of Health, Labour and Welfare. “Hoken iryou jouhou bunnya no hyoujun kikaku (kousei roudou syou hyoujun kikaku) ni tuite” no ichibu kaisei ni tsuite (Partial revision of “the standards in the field of health and medical information (the national standards by the Ministry of Health, Labour and Welfare of Japan).”); 2021 (in Japanese). <http://helics.umin.ac.jp/files/MhlwTsuuchi/MhlwTuuchi_20210326.pdf> (Accessed October 12, 2021).

[15] OpenCV team. Open CV. <https://opencv.org/> (Accessed October 12, 2021).

[16] Japan Association for Medical Informatics Healthcare Information Technologist Certification. Iryo joho gijutsushi ikusei totatsu mokuhyo (Healthcare Information Technologist : GIO/SBO); 2016 (in Japanese). <https://www.jami.jp/jadite/new/first/toutatsu-f.html> (Accessed September 2, 2021).

[17] Japan Association for Medical Informatics Healthcare Information Technologist Certification. Jokyu iryo joho gishi no ippan mokuhyo oyobi kodo mokuhyogun (GIO/SBOs) ver.1.1 (Senior Healthcare Information Technologist: GIO/SBO ver.1.1); 2017 (in Japanese). <https://www.jami.jp/jadite/new/senior/toutatsu-s.html> (Accessed September 2, 2021).

[18] Wood EJ. Problem-based learning: Exploiting knowledge of how people learn to promote effective learning. Bioscience Education 2004; 3: 1–12.

[19] Bay M, Staver JR, Bryan T, Hale JB. Science instruction for the mildly handicapped: Direct instruction versus discovery teaching. Journal of Research in Science Teaching 1992; 29: 555–570.

[20] Cyr AA, Anderson ND. Trial-and-error learning improves source memory among young and older adults. Psychol Aging 2012; 27: 429–439. 21859216 10.1037/a0025115

